# Supplementary material for: Mixed Tree Nut Snacks Compared to Refined Carbohydrate Snacks Resulted in Weight Loss and Increased Satiety during Both Weight Loss and Weight Maintenance: A 24-Week Randomized Controlled Trial
Source: Nutrients. 2021 Apr 30;13(5):1512. doi: 10.3390/nu13051512 (PMC8145615; doi:10.3390/nu13051512)
Supplement: Supplementary file 1 [file nutrients-13-01512-s001.zip › nutrients-1160702-supplementary.pdf]

Supplementary Materials

Supplementary Table S1: Reason for early withdrawal from the study.

Reasons for leaving the study	Treenuts (No. of participants)	Pretzel (No. of participants)
Patient will not be able to continue the study due to other commitments	1	0
Patient did not respond to emails and phone calls	3	8
Patient not be able to continue the study due to personal issues	3	5
Withdrawn from the study due to not be able to eat Nuts every day	1	0
Withdrawn from the study due to not be able to eat Pretzels every day	0	1
Patient decided to withdraw. No reason given	1	3
Patient move out of state	1	1
Patient dropped the study due to headaches	1	0
Patient became pregnant	0	1
Patient decided to withdrawn from the study due to traveling and many activities	0	1
Not be able to continue due to busy work schedule	0	1
Withdrawn by the investigator due to gift card issue	0	1
Busy schedule , unable to finish study	0	1

Supplementary Table S2: Effect of tree nuts and pretzels on body weight and body mass index (BMI).

	Treenut		Pretzel	
Time (wk)	Weight (kg)	BMI (kg/m^2)	Weight (kg)	BMI (kg/m^2)
0	87.2±1.7	31.1±0.35	83.4±2.2	30.6±0.39
4	86.1±1.7**	30.7±0.36**	82.3±2.2**	30.2±0.44**
12	85.6±1.7**	30.5±0.37**	81.5±2.3**	29.7±0.55**
24	85.7±1.7**	30.5±0.37**	82.0±2.3**	30.1±0.51**

Data are means±SEM. **P<0.0001 compared to baseline in the same treatment group. Linear Mixed effects model was used to analyze the repeated measurements within subjects and to evaluate the change within and between tree nut and pretzel control group. Tree nuts N=56, Pretzel N=39.

Supplementary Table S3: Effect of tree nuts and pretzels on serum hs-CRP, MCP-1, C, IL-10 and TNF-α concentrations.

	Treenut				Pretzel			
Time (wk)	IL-10 pg/ml	MCP1 pg/ml	TNFα pg/ml	hs-CRP µg/ml	IL-10 pg/ml	MCP1 pg/ml	TNFα pg/ml	hs-CRP µg/ml
0	5.3±0.9	298±13#	8.1±0.6	15.6±2.5	10. ±5.2	258±18	7.5±0.8	14.4±2.9
4	5.7±0.8	278±12*	8.3±0.6	13.2±1.9	8.9±5.0	262±19	8.0±0.7	12.9±2.0
12	5.5±0.9	297±14	8.3±0.7	17.6±2.2	8.0±4.1	267±20	7.7±0.7	13.6±3.0
24	5.2±0.7	291±13	7.8±0.5	17.2±2.7	8.1±4.0	274±18	7.7±0.6	15.0±3.3

Data are means±SEM. * P<0.05, compared to baseline in the same treatment group. # P<0.05, comparing tree nut to pretzel group. Linear Mixed effects model was used to analyze the repeated measurements within subjects and to evaluate the change within and between tree nut and pretzel control group. Tree nuts N=56, Pretzel N=39. Hs-CRP, high sensitive C-reactive protein; MCP-1, monocyte chemoattractant protein-1; IL-10, interleukin 10 and TNFα, tumor necrosis factor alpha.
